# Genome cloning and genetic evolution analysis of eight duck-sourced novel goose parvovirus strains in China in 2023

**DOI:** 10.3389/fmicb.2024.1373601

**Published:** 2024-05-02

**Authors:** Guangjian Zhan, Nan Liu, Xiaole Fan, Wansi Jiang, Mengxue Yuan, Yunwang Liu, Shishan Dong

**Affiliations:** ^1^College of Veterinary Medicine & Traditional Chinese Veterinary Medicine, Hebei Agricultural University, Baoding, Hebei Province, China; ^2^Xintai Jianyuan Breeding Poultry Limited Company, Taian, Shandong Province, China; ^3^Jiangsu Yike Food Group Limited Company, Suqian, Jiangsu Province, China; ^4^College of Animal Science and Veterinary Medicine, Shandong Agricultural University, Tai’an, Shandong Province, China

**Keywords:** Cherry Valley duck, novel goose parvovirus, short beak and dwarfism syndrome, mutation site, phylogenetic analysis

## Abstract

**Introduction:**

There are three major categories of waterfowl parvoviruses, namely goose parvovirus (GPV), Muscovy duck parvovirus, and novel goose parvovirus (NGPV). NGPV can infect both Cherry Valley ducks and mule ducks, resulting in short beaks and dwarfism syndrome, and the incidence of short beaks and dwarfism syndrome rises annually, posing a significant threat to the waterfowl breeding and the animal husbandry. Therefore, clarifying the biological characteristics and genetic evolution of NGPV is very important for the prevention and control of NGPV.

**Methods:**

Ducks with short beaks and dwarfism syndrome from Shandong and Henan Province were investigated by dissection and the tissue samples were collected for study. The NGPV genome was amplified by PCR, and the genome was analyzed for genetic evolution.

**Results:**

Eight strains of NGPV were isolated, which were designated as HZ0512, HZ0527, HZ0714, HZ0723, HZ0726, HZ0811, HZ0815, and HN0403. The nucleotide homology among these strains ranged from 99.9% to 100%. The eight strains, along with other NGPVs, belong to GPV. The eight strains showed a 92.5%–98.9% nucleotide homology with the classical GPV, while a 96.0%–99.9% homology with NGPV.Therefore, it can be deduced that there have been no major mutations of NGPV in Shandong and Henan provinces in recent years.

**Discussion:**

This study lays a theoretical foundation for further studying the genetic evolution and pathogenicity of NGPV, thereby facilitating the prevention and control of NGPV.

## Introduction

Waterfowl parvoviruses comprise Muscovy duck parvovirus (MDPV), goose parvovirus (GPV), and novel goose parvovirus (NGPV). These viruses exhibit similar morphological structures and physical characteristics, including a 5-kb linear genome and a non-enveloped 20-sided capsid with a diameter of 20–22 nm. The viral genome consists of two major open reading frames (ORFs) responsible for encoding nonstructural (NS) proteins and capsid proteins (VP). Notably, capsid proteins play a crucial role in determination of pathogenicity and host range (Tu et al., 2015). In addition, VP proteins can be divided into VP1, VP2, and VP3, among which VP3 is the most expressed and tunable protein among capsid proteins, and can induce antibody neutralization and provide protective immunity for waterfowl (Lian et al., 2020). The peptide chain length of VP1 is longer than that of VP2 and VP3, and the entire amino acid sequences of VP2 and VP3 are encompassed in the carboxyl terminal part of VP1 (Tsai et al., 2004).

**Figure 1:**
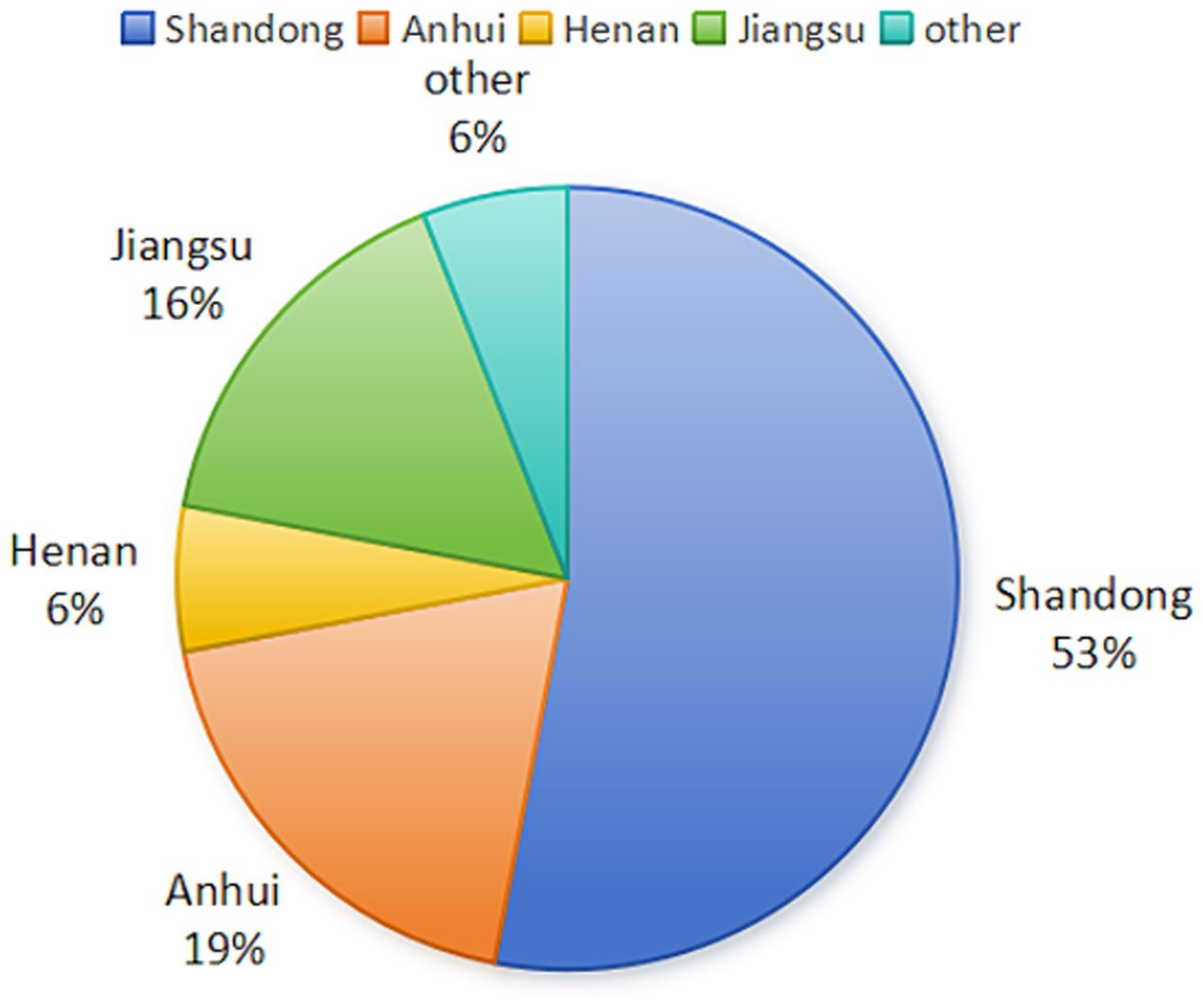
Distribution of NGPV cases in China (Data source: NCBI database).

Two major outbreaks of novel goose parvovirus were reported in Taiwan, China during 1982–1989 (Li et al., 2021). In 2015, an outbreak of “short beak and dwarfism syndrome” (SBDS), which is characterized by symptoms such as short beaks, exposed tongues, stunted growth, weak bones, and diarrhea, was reported in Cherry Valley duck flocks in China. This syndrome, first reported in the 1900s, was also observed in French mule ducks in the 1970s (Palya et al., 2009; Fu et al., 2017). The disease has an incidence rate of 10–30% and a mortality rate of 2–6%, posing a significant threat to waterfowl breeding in China (Li et al., 2018). In recent years, 53% of NGPV cases were found in Shandong, 19% in Anhui, 6% in Henan, 16% in Jiangsu, and 6% in other provinces of China (Figure 1). NGPV has been identified as the primary pathogen of this disease. NGPV is a newly discovered waterfowl parvovirus with significant differences from GPV and MDPV. GPV can infect geese and Muscovy ducks, leading to a condition known as Derzsy’s disease, and MDPV primarily infects Muscovy ducks (Glávits et al., 2005; Ji et al., 2010). Nevertheless, NGPV can infect both Cherry Valley ducks and mule ducks, resulting in SBDS. To make it worse, NGPV can co-infect with duck circovirus and duck hepatitis virus.

To clarify the biological characteristics of NGPV and its genetic evolution in specific regions of China, we isolated the strains and identified the pathogen and sequence the genomes of eight diseased Cherry Valley broiler ducks with typical SBDS symptoms. These ducks were obtained from Shandong and Henan Province. We conducted a comparative analysis of the sequences and phylogenetic development involving 47 reference strains, which allowed the elucidation of the genetic variability of NGPV in Shandong and Henan and offered valuable insights for the control and prevention of SBDS. Additionally, this study lays a foundation for further studying the genetic evolution and pathogenicity of NGPV.

## Materials and methods

### Viral materials and DNA extraction

Eight groups of 28-day-old Cherry Valley ducks from Shandong and Henan with typical SBDS symptoms, including ruffled feathers, stunted growth, shortened beaks, swollen tongues, and diarrhea, were enrolled. Dissection revealed swollen tongues, kidney hemorrhaging, and enlarged gallbladders in some of the sick ducks, while no significant lesions were observed in other organs. Laboratory primers were used for avian influenza virus, novel duck reovirus, Duck Tembusu virus, duck circovirus, duck astrovirus, and NGPV identification in PCR, wherein all samples were tested positive for NGPV, while the other viruses yielded negative results. The sequences of primers used for identification are shown in Table 1. These groups were denoted as HZ0512, HZ0527, HZ0714, HZ0723, HZ0726, HZ0811, HZ0815, and HN0403, respectively. Liver and spleen tissue samples were collected and stored at −80°C.

**Table 1:** Primer sequence for identification.

Primers	Sequence
Avian influenza virus-F	TTCTAACCGAGGTCGAAAC
Avian influenza virus-R	AAGCGTCTACGCTGCAGTCC
Novel duck reovirus-F	TGAGACGCCTGACTACGATT
Novel duck reovirus-R	ATGCTTGGAGTGAGACGACT
Duck Tembusu virus-F	TGCATGGGTCAATCGATC
Duck Tembusu virusR	ACTGTTCAAGGCTGCTGT
Duck circovirus-F	TTGAAGAGTCGCTGGGAGGAA
Duck circovirus-R	CTTAGCAACAAACTGGGTCA
Duck astrovirus-F	TTACCAACTGGGGAGGTTTG
Duck astrovirus-R	CCATCACTCCTTTTAACCAACG
NGPV-F	GAGCATCAACTCCCGTATGTCC
NGPV-R	CTACTTCCTGCTCGTCCGTGA

The preserved tissue samples were diluted and homogenized with 0.9% NaCl, followed by centrifugation at 12,000 rpm for 60 s at room temperature. The supernatant was collected. Additionally, viral genomic DNA extraction was conducted using the DNA extraction kit (Bio Flux, Hangzhou, China).

### Primer design

Five pairs of primers were designed to amplify the NGPV gene based on the primers described in the article and the NGPV genome sequence available in GenBank (Ge et al., 2017). The primer sequences are shown in Table 2.

**Table 2:** Primers used to amplify the genome of NGPV.

Primers	Sequence
NGFP-F1:	CTYATTGKAGGGTTCGTTCGTTCG
NGFP-R1:	GCATGCGCGTGGTCAACCTAACA
NGFP-F2:	CGCGCGGTCAGCCMWATAGTT
NGFP-R2:	AGGGTACAGCATGGACAATAG
NGFP-F3:	AAGCAACCTGAACTGCAGTGGGCC
NGFP-R3:	GAGGGGCTCCAGCTTTCAGATTCC
NGPV-F4:	TGACGATGCTGAAAATGAACA
NGPV-R4:	GTCCTGTGAATGAGCGAACA
NGPV-F5:	GTTCCCGTCGGATGTCTATG
NGPV-R5:	ACGAACCCTCCAATAAGACTCA

### Amplification of the genome of NGPV

The gene sequence of NGPV was amplified using the Taq PCR Master Mix Kit. For each reaction, 2 μL of extracted DNA, 10 μL of the Mix, 6 μL of H2O, and 1 μL of upstream and downstream primers each were added. For DNA amplification, denaturation at 95°C for 4 min was conducted, followed by denaturation at 95°C for 15 s, annealing at 57°C for 15 s, and extension at 72°C for 15 s; the extension was repeated for a total of 35 times, followed by a final extension at 72°C for 5 min. Subsequently, 10 μL of the PCR product was subjected to 1% agarose gel electrophoresis. The positive PCR products were then extracted from the gel and sent to Beijing Prime Biotechnology Co., Ltd. for sequencing. The sequencing results were analyzed by DNA Star (Version 7.1.0, Madison, United States).

### Genome sequence analysis

Nucleotide and amino acid sequences were aligned using the Clustal W method in Megalin 7.1.0 (DNAStar Inc., Madison, United States). MEGA7 was employed to compare the gene sequences of the eight NGPV strains with the reference strains. Phylogenetic trees were developed using the Neighbor-joining method based on the aligned nucleotide sequences of NGPV strains. A total of 1,000 bootstrap replicates were involved. Sopma^1^ was employed to analyze the secondary structures of NS and VP proteins, and the Swiss-Model^2^ was employed to develop a model of the tertiary structure of VP proteins. The gene sequences used in this study are presented in Table 3.

**Table 3:** Information of the strains used in this study.

GenBank accession no.	Strain name	Place of origin	Host	Genome size (bp)	Virus
EU583392	VG32/1	China, 2008	Goose	5,104	GPV
JF926697	P	China,1988	Muscovy duck	4,265	MDPV
HQ891825	GDaGPV	China, 1978	Goose	5,106	GPV
KC171936	SAAS-SHNH	China, 2012	Muscovy duck	5,061	MDPV
KC184133	E	China, 2012	Goose	5,125	GPV
KC996730	YZ99-6	China, 1999	Goose	5,046	GPV
KM093740	GX5	China, 2011	Muscovy duck	5,132	MDPV
KM272560	LH	China, 2012	Goose	5,047	GPV
KR075690	FJM3	China, 2013	Muscovy duck	5,017	MDPV
KR091960	YZ	China, 2013	Goose	5,049	GPV
KR136258	Yan-2	China, 2013	Goose	5,106	GPV
KT751090	QH15	China, 2015	Cherry Valley duck	5,048	GPV
KT865605	FZ91-30	China, 1991	Muscovy duck	5,131	MDPV
KT935531	JS1	China, 2015	Cherry Valley duck	5,106	NGPV
KU641558	CVSD01	China, 2015	Cherry Valley duck	5,106	GPV
KU684472	GER	Poland,2015	Ornamental duck	4,792	GPV
KU844281	P	China, 1988	Muscovy duck	5,123	MDPV
KU844282	P1	China, 2015	Muscovy duck	5,118	MDPV
KU844283	FJ/15	China, 2015	Mule duck	5,030	NGPV
KX000918	YY	China, 2000	Muscovy duck	5,075	MDPV
KX384726	DS15	China,2015	Cherry Valley duck	5,104	NGPV
KY475562	RC16	China,2015	Goose	5,046	GPV
KY679174	SC16	China,2016	Cherry Valley duck	5,109	NGPV
MF441221	SDLY1512	China,2015	Cherry Valley duck	5,054	NGPV
MF441222	SDLY1602	China, 2016	Cherry Valley duck	5,110	NGPV
MF441223	SDHZ1604	China, 2016	Cherry Valley duck	5,054	NGPV
MF441224	SDDY1605	China, 2016	Cherry Valley duck	5,054	NGPV
MF441225	AH1606	China, 2016	Cherry Valley duck	5,054	NGPV
MF441226	JS1603	China,2016	Cherry Valley duck	5,055	NGPV
MF441227	AH1605	China,2016	Cherry Valley duck	5,054	NGPV
MH209633	DY16	China, 2016	Goose	5,046	GPV
MH444513	AH	China, 2018	Cherry Valley duck	5,053	NGPV
MH444514	GD	China,2016	Mule duck	5,106	NGPV
MH807697	JH06	China, 2006	Muscovy duck	5,071	MDPV
MH807698	JH10	China, 2010	Muscovy duck	5,071	MDPV
MK000549	HuN001	China, 2018	Linwu duck	5,110	NGPV
MK737642	HN1P	China, 2019	*Anas platyrhynchos*	5,058	NGPV
MN356044	SDJN19	China, 2019	Cherry Valley duck	5,054	NGPV
MN549532	GDSG1901	China, 2019	Domestic partridge duck	5,109	NGPV
MN549533	GDQY1802	China, 2019	Domestic partridge duck	5,109	NGPV
MT646163	AHAU41	China, 2019	Duck	5,057	GPV
MT646164	AHAU30	China, 2019	Duck	5,039	NGPV
MW386077	Corum/19	Turkey, 2019	*Anser anser* domesticus	4,709	GPV
MW386078	Konya/19	Turkey, 2019	*Anser anser* domesticus	4,709	GPV
MW386079	Yozgat/19	Turkey, 2019	*Anser anser* domesticus	4,709	GPV
ON645931	HNXY21	China, 2021	Duck	5,073	NGPV
U25749	B	Hungary, 1995	Goose	5,106	GPV
PP068765	SDHZ0512	China, 2023	Cherry Valley duck	4,643	NGPV
PP068766	SDHZ0527	China, 2023	Cherry Valley duck	4,641	NGPV
PP068767	SDHZ0714	China, 2023	Cherry Valley duck	4,656	NGPV
PP068768	SDHZ0723	China, 2023	Cherry Valley duck	4,641	NGPV
PP068769	SDHZ0726	China, 2023	Cherry Valley duck	4,619	NGPV
PP068770	SDHZ0811	China, 2023	Cherry Valley duck	4,702	NGPV
PP068771	SDHZ0815	China, 2023	Cherry Valley duck	4,623	NGPV
PP068772	HN0403	China, 2023	Cherry Valley duck	4,674	NGPV

## Results

### Virus identification

**Figure 2:**
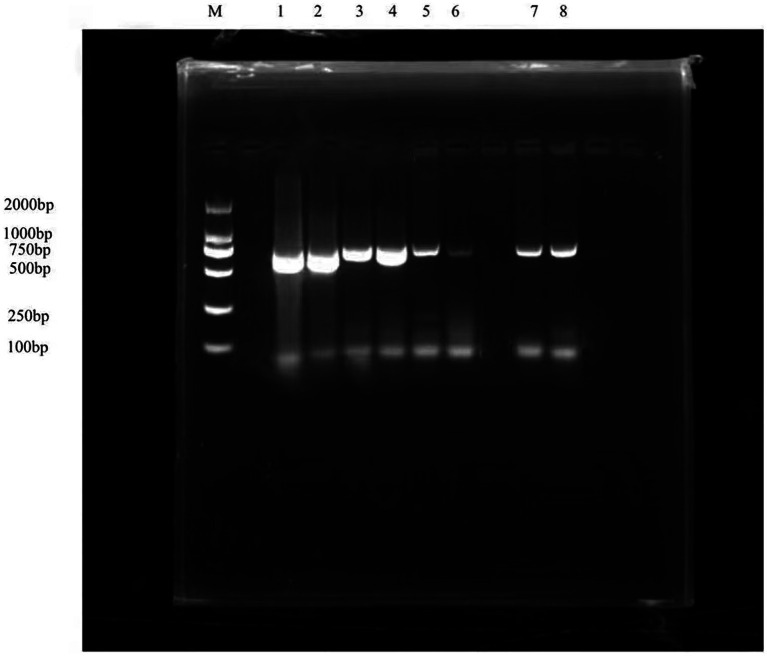
Results of NGPV identification. M: DL 2000 DNA maker, 1–8: Results of NGPV identification (661 bp).

PCR was performed on the sample ducks using the laboratory preserved Avian influenza virus, Novel duck reovirus, Duck Tembusu virus, duck circovirus, duck astrovirus, and NGPV identification primers. The results showed that only NGPV identification primers led to amplified positive bands (Figure 2). The detection rate of NGPV was 25%, among which HZ0403 was detected in ducks from Henan and other strains were detected in ducks from Shandong.

### Genome sequences of the eight NGPV strains

The gene sequences of the eight NGPV strains isolated in this study encompassed the complete coding sequences (CDs) of two major ORFs, which encode NS and VP proteins. The NS genes had a total length of 1884 bp and are responsible for encoding 627 amino acids, while the VP genes had a total length of 2,199 bp and are responsible for encoding 732 amino acids. The eight NGPV strains isolated in this study showed a nucleotide identity of 99.9–100% and an amino acid identity of 99.5–99.9%.

The nucleotide homology of the eight NGPV strains with the MDPV reference strains, GPV nucleotides, and NGPV were 80.8–85.3%, 92.5–98.9% and 96.0–99.9%, respectively.

**Figure 3:**
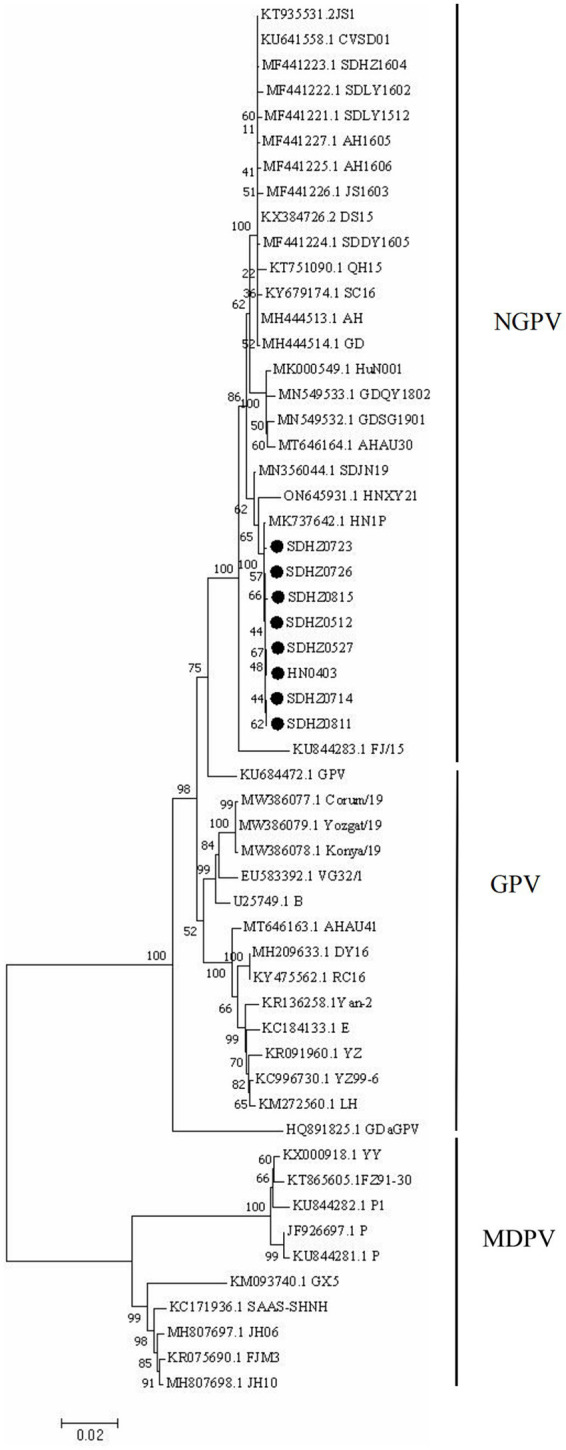
Phylogenetic tree of NGPV strains obtained based on the genome nucleotide sequences. Numbers at nodes indicate bootstrap percentages obtained after 1,000 replicates. Genotypes are indicated on the right side of the trees.

The results of the genome-wide phylogenetic tree analysis (Figure 3) demonstrated that waterfowl parvovirus strains can be categorized into GPV and MDPV. All NGPV strains consisted of a sub-group of the GPV group, and the eight strains isolated belonged to the NGPV group. Notably, they were the most closely related to the HN1P strain isolated from ducks from Henan.

### Amino acid sequences of the nonstructural proteins

**Figure 4:**
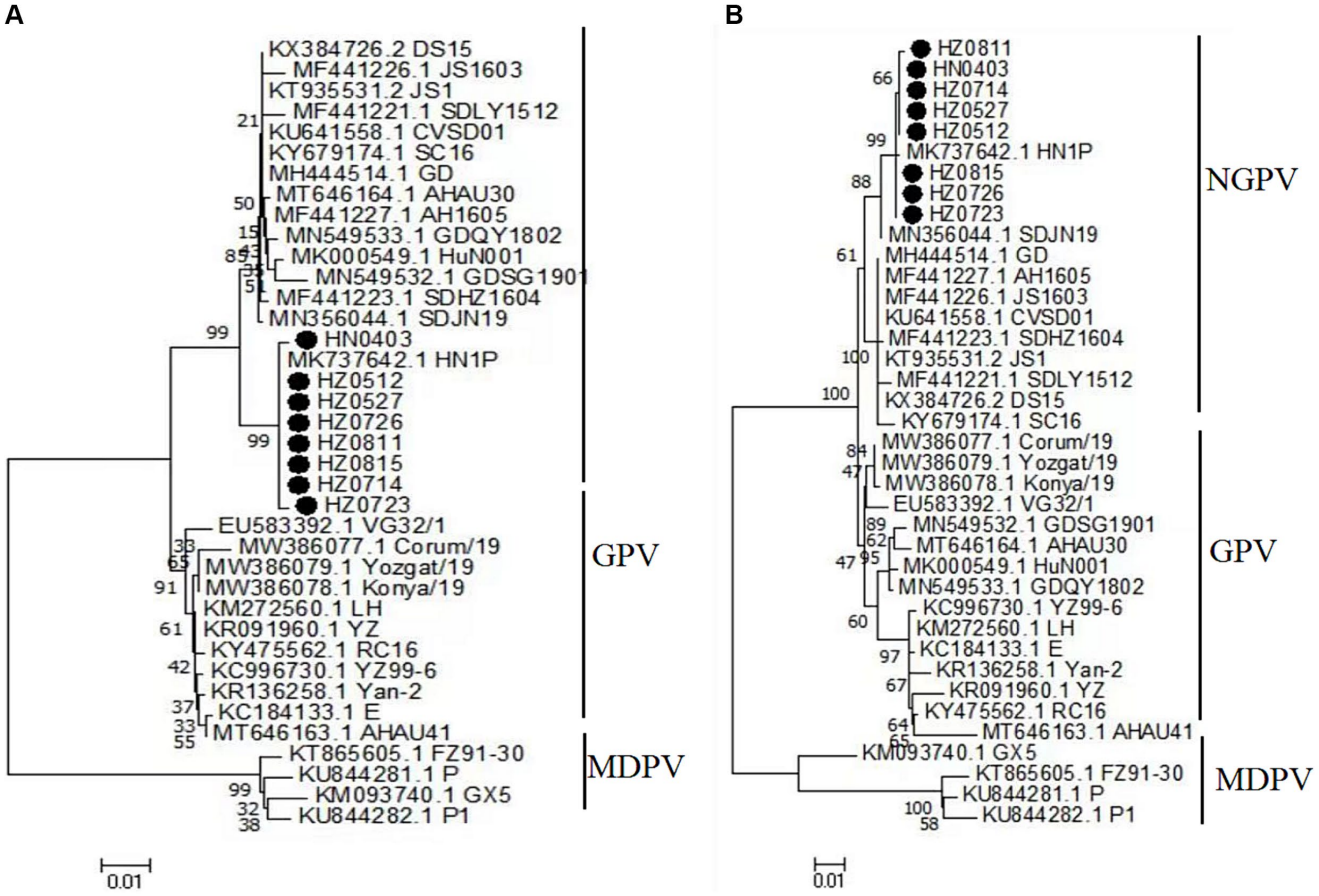
Phylogenetic tree of NS complete amino acid gene **(A)**, phylogenetic tree of VP complete gene amino acid **(B)**. Numbers at nodes indicate bootstrap percentages obtained after 1,000 replicates. Genotypes are indicated on the right side of the trees.

The amino acid sequences of the NS proteins of the eight viruses were determined based on the nucleotide sequences of the NS gene. A phylogenetic tree was established using the amino acid sequences of these proteins and 30 reference strains (Figure 4A). Notably, the amino acid sequences of the NS proteins of the eight viruses were clustered together within the same branch as the NGPV HN1P strain. Compared with the classical GPV vaccine strain, the NS proteins of the isolated strains had 19 common mutation sites, as shown in Table 4.

**Table 4:** The amino acid mutation sites of NS protein of NGPV compared to classical GPV.

Virus	Sites in NS proteins																		
	27	50	123	131	140	159	223	250	350	468	534	553	555	573	575	594	602	608	617
GPV	H	I	M	K	A	L	T	E	A	V	S	R	N	E	M	D	A	K	V
NGPV	Q	T	I	R	S	V	A	D	V	I	P	K	T	K	I	Y	T	I	A

**Figure 5:**
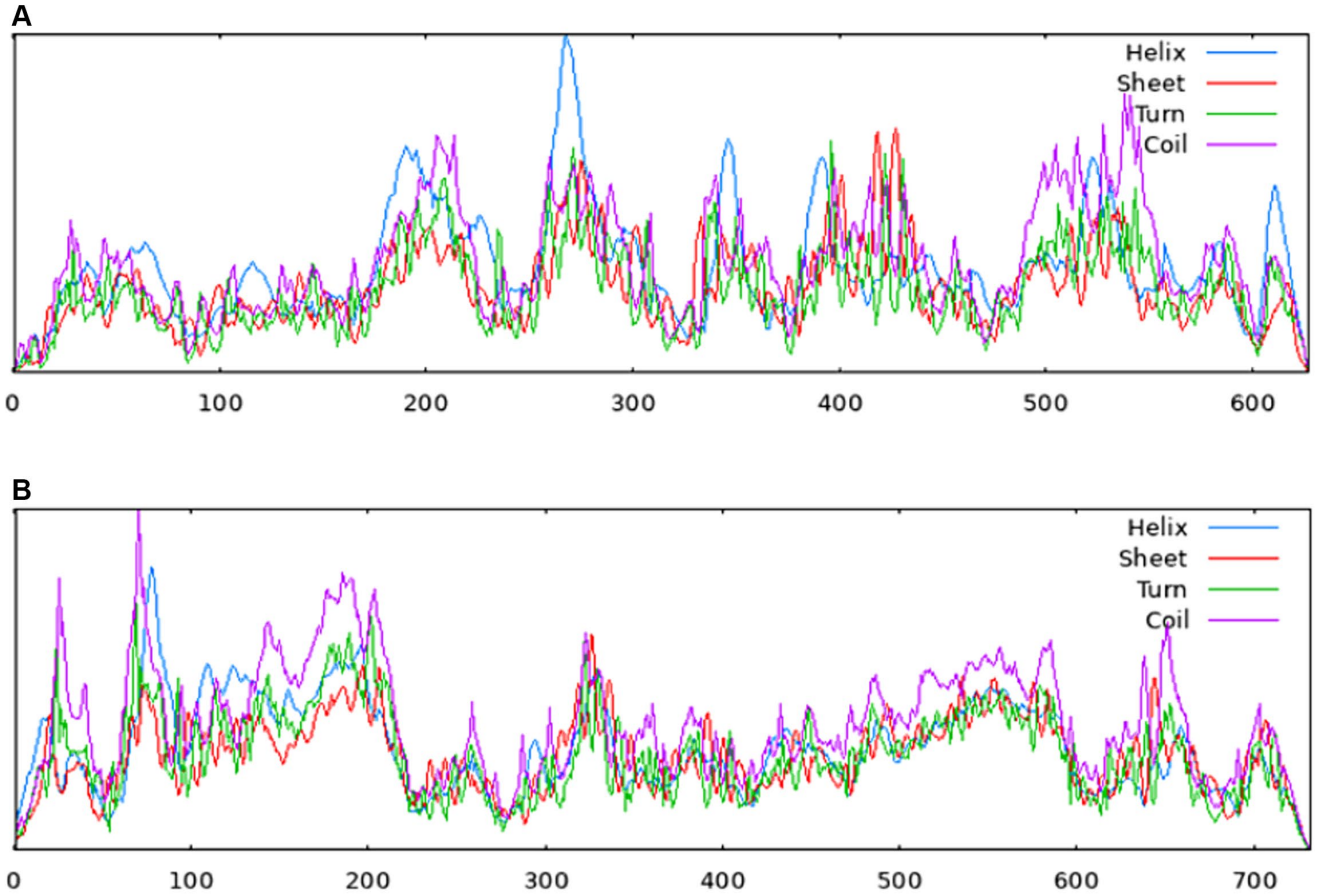
Secondary structures of NS protein **(A)** and VP protein **(B)** predicted by the Sopma. Red indicates sheet, blue indicates helix, green indicates turn and purple indicates coil.

The homology analysis revealed that the amino acid sequences of the NS proteins in the isolates shared 89.2–89.6%, 96.8–97.3%, and 96.7–100% similarities to those of MDPV, GPV and NGPV, respectively. Within the eight NGPV isolates, the homology of amino acids of the NS proteins ranged from 99.7 to 100%. Due to the high genomic similarity of NGPV isolates, the NS and VP proteins of the eight NGPV isolates showed negligible differences in secondary structure. The results of Sopma showed that the NS protein was composed of helix (39.71%), sheet (12.76%), turn (3.99%) and coil (43.54%) (Figure 5A).

### Amino acid sequences of the capsid proteins

**Figure 6:**
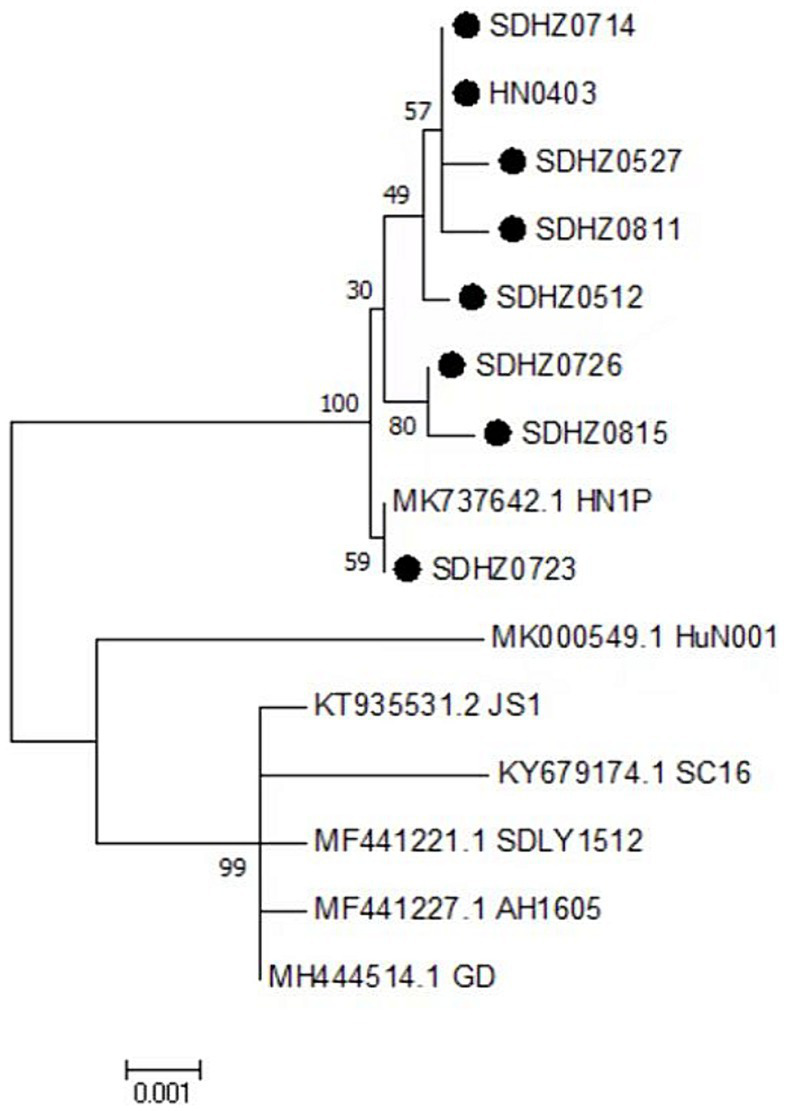
Phylogenetic tree of VP3 complete nucleotide sequence. Numbers at nodes indicate bootstrap percentages obtained after 1,000 replicates. Genotypes are indicated on the right side of the trees.

The amino acid sequences of the VP proteins of the eight viruses were determined based on the nucleotide sequence of the VP gene. Then, a phylogenetic tree was established by aligning these amino acid sequences based on the 30 reference sequences (Figure 4B). All isolates were within the same branch as the NGPV HN1P strain, and HZ0815, HZ0726, and HZ0723 were clustered in the same branch as the HN1P strain. Moreover, the eight isolates were not in the same branch with the classic vaccine strain VG32/1 (EU583392). A comparison of the VP proteins of the isolates and the classical GPV vaccine strains revealed 12 common mutation sites, seven of which were in the VP3 region (198–732 aa), as shown in Table 5. Additionally, nucleotide sequences of VP3 of the isolates and 7 reference strains were compared, and phylogenetic trees were established. The results showed that all isolates were only in the same branch as HN1P though their homology exceeded 98.7% (Figure 6).

**Table 5:** The amino acid mutation sites of VP protein of NGPV compared to classical GPV.

Virus	Sites in capsid proteins											
	75	89	93	116	142	261	450	479	485	520	534	584
GPV	N	Q	A	Q	D	S	S	R	T	T	I	I
NGPV	K	L	S	Y	E	A	N	Q	S	A	S	T

**Figure 7:**
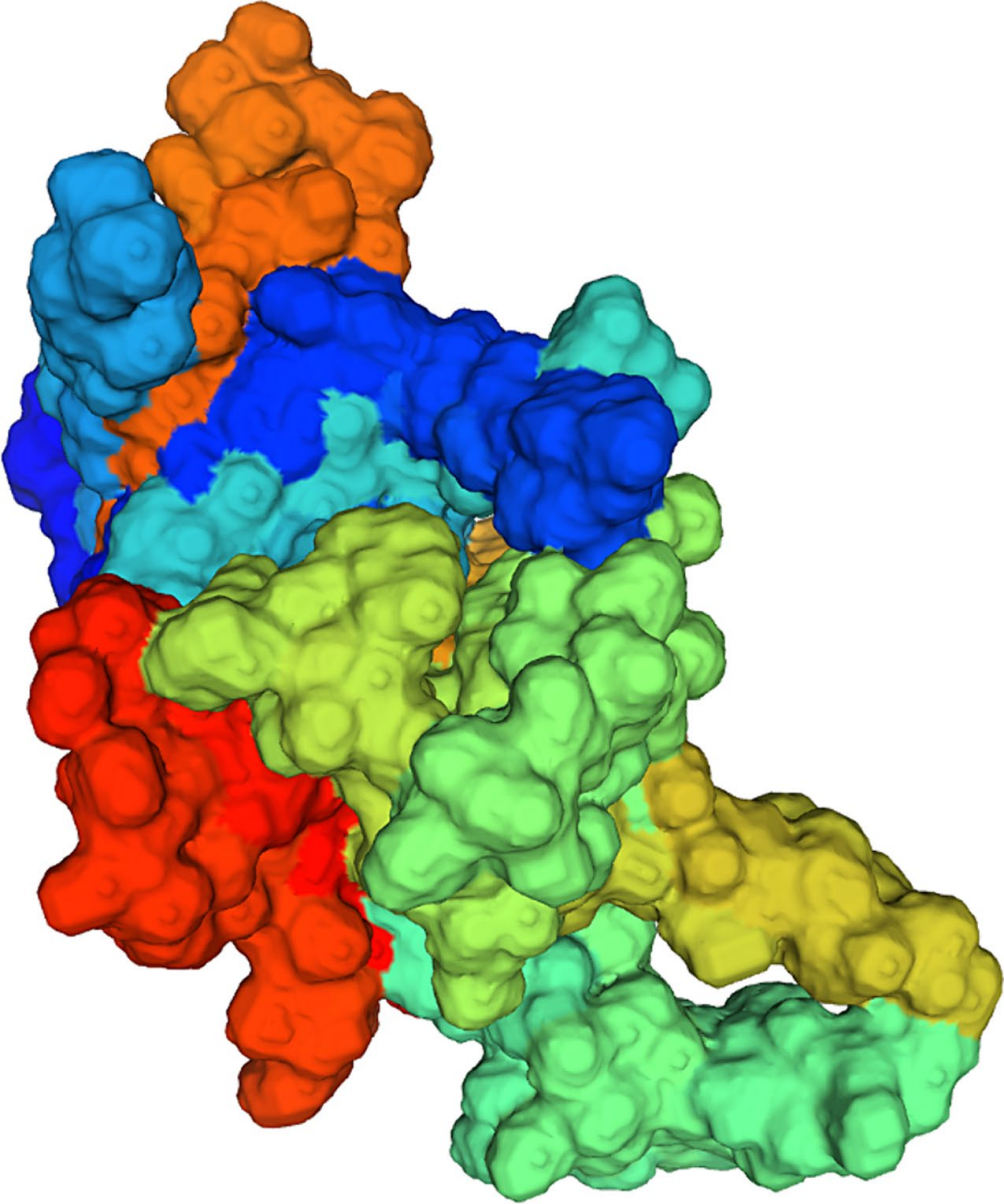
Model establishment by the Swiss Model. The VP protein of HZ0512 appears as a surface form.

The homology of amino acid sequences of the VP proteins of the isolates ranged from 99.7 to 100%. The isolates showed 87.3–90.8%, 94.8–98.2%, and 97.1–99.9% homologies to the MDPV, GPV, and NGPV reference strains, respectively. Determination of the secondary structure by Sopma demonstrated that the NS protein was composed of helix (13.39%), sheet (15.98%), turn (2.32%) and coil (68.31%) (Figure 5B). Swiss-Model was used for template searching and model establishment. As indicated, the VP protein of HZ0512 had the highest similarity to Hum8 capsid (59.84%), while 1-214aa had a low similarity to Hum8 capsid, and its tertiary structure could not be determined in this case. The 215–732 aa model was developed with 7ltm as the template (Figure 7).

## Discussion

Waterfowl parvovirus is a highly contagious and fatal pathogen affecting goslings and ducklings. It belongs to the species of Anseriform dependoparvovirus 1, the genus of Dependoparvovirus. Waterfowl parvovirus is classified as a vesiculovirus. It has a small icosahedral capsid assembled from 32 capsids, with a diameter of 20–22 nm; it is typically characterized by a single-stranded DNA genome. Its full-length genome contains two open reading frames: the left encoding the non-structural (NS) proteins (NS1 and NS2), and the right encoding the structural proteins (VP1, VP2, and VP3). SBDS was first reported in mule ducks in France during the 1970s. It has also been found in various regions of China, including Shandong, Henan, Guangxi, and Sichuan (Matczuk et al., 2020). NGPV has been confirmed as the causative agent of SBDS (Matczuk et al., 2020). This virus can be transmitted both horizontally and vertically and can co-infect ducks with duck circovirus and duck hepatitis virus, resulting in the feather shedding syndrome (Chen et al., 2015; Matczuk et al., 2020; Yang et al., 2020). Moreover, goose parvovirus is also involved in the angel wing syndrome in Muscovy ducks, and the infected ducks would exhibit SBDS and angel wing symptoms (Sallam et al., 2022). In addition, the influence of NGPV on Cherry Valley ducks has been explored from the perspective of intestinal flora, and it has been found that NGPV could cause an imbalance of intestinal flora, accompanied by increases in cecal short-chain fatty acids (Chen et al., 2021).

Ducks with SBDS exhibit various symptoms, including short bill, protruding tongue, weak bones, and growth retardation. This condition has a prevalence of up to 30% in duck flocks, resulting in a mortality rate of 2–6%. Despite that the mortality is not high, a significant number of infected ducks experience severe growth retardation, resulting in a culling rate of about 80% (Chen et al., 2015). Consequently, SBDS poses a substantial threat to waterfowl breeding in China.

This study investigated eight NGPV strains isolated from Shandong and Henan. These strains contain two major ORFs: encoding VP proteins and encoding NS proteins. These isolates had a nucleotide homology of 100% with each other. Phylogenetic analysis based on reference strains demonstrated that the eight isolates belong to the NGPV, which is consistent with the results of the homology analysis. Additionally, these NGPV isolates were closely related to HN1P, a strain isolated from ducks in Henan, China, with a nucleotide homology of 99.9%.

The NS protein plays a crucial role in viral replication, gene expression, and apoptosis. It facilitates essential activities such as DNA replication and transcription (Brown et al., 1995). The nucleotide sequences encoding the NS protein in the eight isolates were found to have 1884 base pairs, which is consistent with previous studies. Phylogenetic analysis of the amino acid sequences of the NS proteins indicated that these isolates developed a significant branch with the NGPV reference strain. Homology analysis showed a homology of 96.8–97.3% and 96.7–100% with GPV and NGPV, respectively. Notably, the homology of the eight isolates and classical GPV and NGPV had negligible differences, making recombination events possible in our isolates. Compared with classical GPV, 19 common mutation sites were found in NS proteins of the eight isolates, among which 140aa (A-S), 159aa (L-V), and 350aa (A-V) were located near motifs associated with viral transcription (Shien et al., 2008). Previous studies have shown that the epitope of GPV NS protein is mainly located in the C-terminal (485–627aa) (Li et al., 2018). Compared with classical GPV, nine amino acid mutations were found at the C-terminal of the NS protein in NGPV. Hence, it can be speculated that the short beak symptoms and “angel wing” symptoms of ducks may be related to mutations in NS proteins.

VP proteins are involved in the synthesis of viral particle capsid proteins, assembly, and release of progeny viruses, and serve as ligands that bind to receptors on the surface of host cells, which is a critical factor in the viral infection process (Tu et al., 2015). About 80% of the VP proteins are VP3, which is the main capsid protein and protective antigen of the virus (Shen et al., 2015; Li et al., 2016). VP3 can induce antibody neutralization and provide immunity towards other waterfowl parvoviruses. It is also a prime candidate antigen for vaccine development and serum diagnostic testing. It has been demonstrated that virus-like particles based on NGPV VP2 can protect ducks from NGPV (Shang et al., 2023). The homology between the eight isolated NGPV and the reference MDPV was 87.3–90.8%; the homology between GPV and GPV was 94.8–98.2%; and the homology between NGPV and NGPV was 97.1–99.9%. The amino acid sequences of VP proteins in the isolates had a 99.9% homology with the NGPV reference strain. Therefore, it can be speculated that VP proteins are exposed to negligible variations in order to guarantee the stability of viral infection. Indeed, amino acids located at the receptor binding site of VP proteins play an important role in the host range and pathogenicity of viruses (Tu et al., 2015). Compared with classical GPV, 12 common mutation sites of VP proteins were found in the isolates, and the change of VP protein in NGPV may be related to host transfer. The seven linear immunodominant epitope fragments of GPV included 35–71aa, 123–198aa, 423–444aa, 474–491aa, 531–566aa, and 616–669aa (Yu et al., 2012). Four out of the 12 amino acid mutations in NGPV are present in these epitopes, and these mutation sites may affect the immunogenicity of NGPV.

Currently, isolation of NGPV from tissues of infected ducks remains challenging, while the incidence of SBDS rises annually, posing a significant threat to the waterfowl breeding and the animal husbandry. Given the widespread prevalence of NGPV, addressing this issue requires a two-fold approach. First, feeding management practices shall be enhanced and standards of environmental sanitation and disinfection shall be strictly followed. Second, development of NGPV vaccines shall be accelerated. While classical GPV vaccines have been reported to offer partial prevention against NGPV, they are at risk of becoming ineffective due to virus mutations. Our results indicated that the eight NGPV isolates have relatively low mutation levels, suggesting a relatively stable outbreak.

## Data availability statement

The datasets presented in this study can be found in online repositories. The names of the repository/repositories and accession number(s) can be found in the article/supplementary material.

## Ethics statement

The animal study was approved by Shandong Agricultural University Animal Care and Use Committee. The study was conducted in accordance with the local legislation and institutional requirements.

## Author contributions

GZ: Formal analysis, Investigation, Methodology, Writing – original draft. NL: Data curation, Formal analysis, Software, Validation, Writing – original draft. XF: Data curation, Investigation, Writing – review & editing. WJ: Data curation, Investigation, Writing – review & editing. MY: Investigation, Validation, Visualization, Writing – review & editing. YL: Investigation, Validation, Visualization, Writing – review & editing. SD: Methodology, Project administration, Supervision, Writing – review & editing.

## Footnotes

^1^ https://npsa-prabi.ibcp.fr/cgi-bin/npsa_automat.pl?page=npsa%20_sopma.html

^2^ http://www.expasy.ch/swissmod/SWISS-MODEL.html
